# Artificial-Intelligence-Assisted Discovery of Genetic Factors for Precision Medicine of Antiplatelet Therapy in Diabetic Peripheral Artery Disease

**DOI:** 10.3390/biomedicines10010116

**Published:** 2022-01-06

**Authors:** Chi-Hsiao Yeh, Yi-Ju Chou, Tsung-Hsien Tsai, Paul Wei-Che Hsu, Chun-Hsien Li, Yun-Hsuan Chan, Shih-Feng Tsai, Soh-Ching Ng, Kuei-Mei Chou, Yu-Ching Lin, Yu-Hsiang Juan, Tieh-Cheng Fu, Chi-Chun Lai, Huey-Kang Sytwu, Ting-Fen Tsai

**Affiliations:** 1 Department of Thoracic and Cardiovascular Surgery, Chang Gung Memorial Hospital, Taoyuan 333, Taiwan; yehccl@cgmh.org.tw; 2 College of Medicine, Chang Gung University, Taoyuan 333, Taiwan; yuching1221@cgmh.org.tw (Y.-C.L.); 8801131@cgmh.org.tw (Y.-H.J.); mr5598@cgmh.org.tw (T.-C.F.); 3 Community Medicine Research Center, Chang Gung Memorial Hospital, Keelung 204, Taiwan; 4 Institute of Molecular and Genomic Medicine, National Health Research Institutes, Miaoli 350, Taiwan; yjchou0810@nhri.edu.tw (Y.-J.C.); paul@nhri.edu.tw (P.W.-C.H.); petsai@nhri.org.tw (S.-F.T.); 5 Advanced Tech BU, Acer Inc., New Taipei City 221, Taiwan; vincent.tsai@acer.com (T.-H.T.); Zack.Li@acer.com (C.-H.L.); Linda.Chan@acer.com (Y.-H.C.); 6 Department of Internal Medicine, Division of Endocrinology and Metabolism, Chang Gung Memorial Hospital, Keelung 204, Taiwan; angelang@cgmh.org.tw (S.-C.N.); f22789@cgmh.org.tw (K.-M.C.); 7 Department of Medical Imaging and Intervention, Chang Gung Memorial Hospital, Keelung 204, Taiwan; 8 Department of Physical Medicine and Rehabilitation, Chang Gung Memorial Hospital, Keelung 204, Taiwan; 9 Department of Ophthalmology, Chang Gung Memorial Hospital, Keelung 204, Taiwan; 10 National Institute of Infectious Diseases and Vaccinology, National Health Research Institutes, Miaoli 350, Taiwan; 11 National Defense Medical Center, Department & Graduate Institute of Microbiology and Immunology, Taipei 114, Taiwan; 12 Departments of Life Sciences and Institute of Genome Sciences, National Yang Ming Chiao Tung University, Taipei 112, Taiwan; 13 Center for Healthy Longevity and Aging Sciences, National Yang Ming Chiao Tung University, Taipei 112, Taiwan

**Keywords:** diabetic peripheral artery disease, artificial intelligence, ticagrelor, clopidogrel, CYP2C19, single nucleotide polymorphism

## Abstract

An increased risk of cardiovascular events was identified in patients with peripheral artery disease (PAD). Clopidogrel is one of the most widely used antiplatelet medications. However, there are heterogeneous outcomes when clopidogrel is used to prevent cardiovascular events in PAD patients. Here, we use an artificial intelligence (AI)-assisted methodology to identify genetic factors potentially involved in the clopidogrel-resistant mechanism, which is currently unclear. Several discoveries can be pinpointed. Firstly, a high proportion (>50%) of clopidogrel resistance was found among diabetic PAD patients in Taiwan. Interestingly, our result suggests that platelet function test-guided antiplatelet therapy appears to reduce the post-interventional occurrence of major adverse cerebrovascular and cardiac events in diabetic PAD patients. Secondly, AI-assisted genome-wide association study of a single-nucleotide polymorphism (SNP) database identified a SNP signature composed of 20 SNPs, which are mapped into 9 protein-coding genes (SLC37A2, IQSEC1, WASHC3, PSD3, BTBD7, GLIS3, PRDM11, LRBA1, and CNR1). Finally, analysis of the protein connectivity map revealed that LRBA, GLIS3, BTBD7, IQSEC1, and PSD3 appear to form a protein interaction network. Intriguingly, the genetic factors seem to pinpoint a pathway related to endocytosis and recycling of P2Y12 receptor, which is the drug target of clopidogrel. Our findings reveal that a combination of AI-assisted discovery of SNP signatures and clinical parameters has the potential to develop an ethnic-specific precision medicine for antiplatelet therapy in diabetic PAD patients.

## 1. Introduction

Peripheral artery disease (PAD) affected 202 million people worldwide in 2010 [1]. The prevalence of PAD among adults has been estimated to be 5.8 to 10.7%, 6.5%, and 3.1% to 24% in the United States, Chinese populations [2], and the sub-Saharan African region [3], respectively. PAD, when complicated by foot ulcers and amputation, is one of the most common causes of morbidity and mortality in diabetic patients [4]. Diabetes markedly increases the risk of PAD; furthermore, diabetes also increases the incidence of major adverse cerebrovascular and cardiac events (MACCEs) in PAD patients. For diabetic patients with PAD complications, especially those who have received a PAD revascularization procedure, a standard anti-platelet regimen for the prevention of limb loss and MACCE has not yet been established.

Evidence proves that dual anti-platelet therapy (DAPT) can be more effective in reducing the rate of ischemic vascular events, target lesion revascularization [5], and post-revascularization MACCE [6] among PAD patients compared to patients receiving monotherapy with aspirin [7]. Although DAPT might be beneficial in cases where there is an extremely high risk of atherosclerosis complications [8,9], it is not recommended as a first-line treatment. Clopidogrel is one of the most widely used anti-platelet medications to inhibit platelet aggregation and thrombus formation [10]. However, there have been heterogeneous outcomes when clopidogrel [10] is used to prevent cardiovascular events among diabetic PAD patients. Several possibilities might explain clopidogrel’s heterogeneous efficacy. Firstly, the available major studies of anti-platelet regimens involving PAD were extrapolated from trials of patients undergoing percutaneous coronary intervention [11,12], and these had limited ethnic diversity and specificity. Secondly, ethnic-specific polymorphisms of enzymes involved in clopidogrel metabolism seem to be associated with a large variation in clopidogrel efficacy among patients with multiple risk factors for cardiovascular disease [13]. Specifically, there are different frequencies for the various loss-of-function CYP2C19 alleles when Asian and Caucasian populations are compared, namely 62.0% and 48.0%, respectively; this difference highlights the importance of ethnic-specific pharmacogenomic studies when treating cardiovascular diseases with clopidogrel [14]. Thirdly, it seems that the single-nucleotide polymorphisms (SNPs) currently known to be associated with clopidogrel catabolic enzymes are only able to explain a portion of the high prevalence of clopidogrel resistance, namely up to 65% among PAD patients [15,16]. Notwithstanding the above, it is known that the majority of ethnic-specific SNPs reside in intronic sequences and inter-genic regions, which suggests that much of the ethnic-specific phenotypic differences affecting clopidogrel resistance are likely to be due to the modulation of pathways that regulate the clopidogrel catabolism, rather than involving the protein functions and enzyme activities that directly catabolize the drug [17,18].

Ticagrelor, a cyclo-pentyl-triazolo-pyrimidine, is another oral antiplatelet drug that can reversibly bind to the P2Y12 receptor with greater and more consistent platelet inhibition than clopidogrel [8,19]. Ticagrelor has been shown to be more effective than clopidogrel. It seems that ticagrelor is able to reduce the risk of cardiovascular death, myocardial infarction and/or stroke among patients with acute coronary syndrome [20] and prior myocardial infarction [21]. Previously, a randomized clinical trial has revealed the non-inferiority of platelet function test (PFT)-guided maintenance therapy with clopidogrel or with prasugrel compared to standard therapy for patients with high risk of thrombosis [22]. Furthermore, two other randomized clinical trials have shown that pharmacogenetic testing is able to identify patients with a higher risk of clopidogrel resistance, and in such circumstances, a reduction in high platelet reactivity using prasugrel is able to significantly decrease the risk of cardiovascular death [23,24]. 

Artificial intelligence (AI) has been used to assist the discovery of a range of different genetic factors in humans and has helped the identification of novel SNPs associated with drug response when treating cancer, psychiatric disease, and cardiovascular disease [25,26]. In a previous study, AI machine learning failed to predict the platelet reactivity since very complex non-linear phenomena of platelet reactivity [27]. However, AI machine-learning has potential for defining complex biological processes, especially those involving interactions between the multiple genetic factors and biochemical pathways that contribute to clopidogrel resistance. Furthermore, we want to seek the optimal ethnic-specific DAPT for the mid-term management of diabetic patients with PAD after revascularization. 

Thus, the aims of this study include the followings: (1) to evaluate the feasibility of PFT-guided DAPT of diabetic patients with symptomatic PAD after revascularization; (2) to seek the potential ethnic-specific DAPT for the mid-term management of diabetic patients with PAD after revascularization; (3) to identify, by AI-assisted methods, a novel ethnic-specific SNP signature for predicting clopidogrel resistance in order to gain insights into the potential mechanism(s) underlying drug resistance. 

## 2. Materials and Methods

Study population. The study (ClinicalTrials.gov Identifier: NCT02762864) is a single-blind, single-center, prospective, pilot randomized trial. Forty-five diabetic patients with femoropopliteal artery PAD were recruited from the Northeastern Taiwan Community Medicine Research Cohort (NTCMRC, ClinicalTrials.gov Identifier: NCT04839796). All patients received a first PFT after one month of treatment with aspirin and clopidogrel. Patients in the PFT-guided group who were identified as having clopidogrel resistance then received Ticagrelor (90 mg per day) with Aspirin (75 mg per day) for 36 months. Patients in PFT-guided group without clopidogrel resistance or in the control group received clopidogrel (75 mg per day) with aspirin (75 mg per day) for 36 months. The inclusion criteria were: age > 20 years; clinical evidence of PAD with claudication or critical limb ischemia (ankle-brachial index (ABI) ≤ 0.9 or ≥ 1.2 and Rutherford classification for chronic limb ischemia 3–6) [28]; and magnetic resonance angiographic evidence of > 50% stenosis or occlusion of the superficial femoral or popliteal artery. The exclusion criteria included: acute limb ischemia caused by embolism; hypersensitivity or known contraindications to aspirin, heparin, clopidogrel, or ticagrelor; known hypersensitivity to gadolinium-based contrast media; an unwillingness to carry out the clinical and angiographic controls; patients with severe asthma or chronic pulmonary obstructive disease; patients with severe coagulopathy, a history of intracranial hemorrhage, the presence of severe hepatic failure, or pregnancy/lactation; or patient who were being treated by dialysis. The study of human specimens collected from the NTCMRC in Taiwan was approved by the Institutional Review Board (IRB) of Chang Gung Medical Foundation (201800686B0). The prospective randomized study was approved by the IRB of Chang Gung Medical Foundation (201503436A3). The whole-genome SNP analysis of all participants was approved by the IRB of Chang Gung Medical Foundation (202000077B0A3). Written informed consent was obtained from all of the participants. 

**Figure 1:**
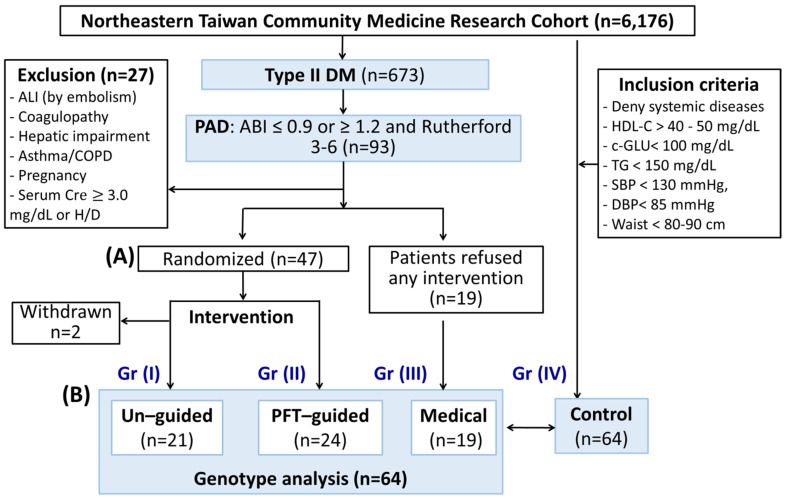
Patient flow diagram. (**A**) Patients were enrolled and prospectively randomized to platelet function test (PFT)-guided or control group. For the PFT group, the age of 20–75 years old, ABI ≤ 0.9 or ≥ 1.2, and Rutherford 3–6 were included. The subjects in the control group were selected according to the following criteria: denied systemic diseases (such as cancer, stroke, diabetes, COPD, CAD, CKD, hypertension, hyperlipidemia), HDL-C > 40 mg/dL (male) or > 50 mg/dL (female), Ac-GLU < 100 mg/dL, TG < 150 mg/dL, systolic blood pressure (SBP) < 130 mmHg and diastolic blood pressure (DBP) < 85 mmHg, and waist circumference < 90 cm (male) or < 80 cm (female). After PAD intervention, patients in the control group received aspirin and clopidogrel for 36 months. Patients with clopidogrel resistance in the PFT-guided group received aspirin and ticagrelor in replacement of clopidogrel. The dosage of ticagrelor was directed by results from their PFT. (**B**) All the genomic DNA of patients enrolled in the study was analyzed and compared to that of age- and sex-match subjects from the Northeastern Taiwan Community Medicine Research Cohort.

The study subjects were divided into the following 4 groups: Post-interventional individuals were randomized in a 1:1 manner into an un-guided group (Group I, Figure 1A) and a PFT-guided group (Group II) using sealed envelopes. Clinical data and clinical samples were also collected from patients who fulfilled the inclusion criteria but refused intervention (Group III). Clinical data and clinical samples from another sixty-four age-matched and sex-matched control participants from the NTCMRC were identified and analyzed to form the control group (Group IV).

Platelet function test (PFT). All patients underwent clinical and Doppler ultrasound follow-up at 1 month after the revascularization procedure and then every 6 months. Platelet function tests to measure P2Y12 receptor inhibition effectiveness were evaluated using the VerifyNow system (VerifyNow^®^ system, Accumetrics^®^, San Diego, CA, USA); this measures platelet reactivity (PRU) in units and was initially carried out one month after clopidogrel therapy began and then every 3 months after the revascularization procedure. Clopidogrel resistance was defined as a patient with a PFT value of >234 PRU [29,30]. In the PFT-guided group, clopidogrel was replaced by ticagrelor, starting at 90 mg per day. The dosage of ticagrelor was titrated according to the PFT results in order to keep the PRU of each patient in the PFT-guided group below 234.

End points. The primary efficacy endpoint was major amputation or unplanned minor limb amputation assessed at 36 months. The secondary key endpoints were death, the occurrence of a major adverse cerebrovascular, and cardiac event (MACCE; this is a composite of all-cause death, hospitalization for acute coronary syndrome, coronary revascularization, or stroke within 3 years after index revascularization procedure), a major bleeding episode (intracranial hemorrhage identified by computer tomography, upper or lower gastrointestinal bleeding require blood transfusion, or access site pseudoaneurysm).

Revascularization procedures. Interventional procedures were performed in accordance with international standards. All patients received both aspirin (100 mg/day) and clopidogrel (75 mg/day) for at least 30 days before the procedure. One bolus dose of sodium heparin (70 units/Kg) was administered at the beginning of the procedure and adjusted according to the activated clotting time (ACT). The ACTs were maintained between 250 and 300 s throughout the whole procedure. At least two orthogonal angiographic projections were acquired in evaluation of the baseline culprit vessel. The stenotic lesion was dilatated with a conventional balloon. A bare metal stent was implanted in the short-segment lesions (<4 cm) located in femoral artery followed by post-dilatation. To treat long-segment chronic total occlusion of the femoral artery, surgical bypass using 6–8 mm of artificial graft implantation was performed. Patients with clopidogrel resistance who had been randomized into the PFT-guided group who received 90 mg of ticagrelor immediately after the procedure, followed by 90 mg per day thereafter. Patients who had been randomized into the PFT-guided group without clopidogrel resistance or who were members of the control group continued being treated with aspirin 100 mg and clopidogrel 75 mg per day. Blood samples for the platelet function tests were taken 2–3 h after last antiplatelet dose prior the procedure and at every 3-month follow-up visit thereafter. 

Sample size calculation. Assuming an expected rate of major amputation or MACCE of around 70% in the un-guided group and of 30% in the PFT-guided group [31,32,33,34], together with a 5% α risk and 20% β risk, 46 evaluable patients (23 in each group) were required. Thus, 54 patients were included to allow for a maximum misclassification and 15% subject loss at 3 years.

Genomic DNA isolation. Peripheral blood was drawn from the subjects using EDTA-coated vacuum tubes. The blood samples were centrifuged at 3000 rpm for 10 min at 4 °C to separate the plasma and white blood cells (WBC). Genomic DNA was isolated from the WBC of each subject using proteinase K digestion followed by phenol/chloroform extraction. Total genomic DNA was precipitated by adding 1/10 volume of 5 M NaCl and 2 volume of 100% ethanol. Finally, the precipitate of genomic DNA was washed by 75% ethanol, dissolved in H_2_O, and stored at 4 °C.

Cytochrome P450 genotype. Whole-genome sequencing was performed to an average depth of 30× using 150 bp paired-end reads on a NovaSeq 6000 platform (Illumina Inc., San Diego, CA, USA). WGS raw data were aligned to GRCh38 using the DRAGEN (Dynamic Read Analysis for GENomics) Bio-IT Platform version 3.6.3 germline pipeline [35]. Sequence alignment data were in the CRAM file format. The CRAM files were used as input for Aldy (v3.0), and this then called the genotypes of various pharmacogenes and star alleles [36], including CYP2C19, CYP3A4, CYP3A5, CYP2B6, and CYP2C9.

Whole-genome SNPs. To identify single-nucleotide polymorphisms (SNPs), we genotyped genomic DNA using the AxiomTM Genome-Wide TWB 2.0 array plate [37], which included 686,463 SNPs. Genotyping analyses were performed on the 64 subjects with DM with PAD complications, as well as the 64 age-matched and sex-matched control subjects without any systemic diseases, all from the NTCMRC. SNPs with a minor allele frequency rate of 0 or a SNP missing rate larger than 10% were excluded from the analysis. In the end, there were 392,885 SNPs available for further analysis.

AI-assisted discovery of SNP signatures. To identify significant SNPs related to clopidogrel resistance, we had two steps in feature selection. The first step is to keep SNPs with odds ratio values > 1 with significant *p*-value. Second, those significant SNPs in the first step were ranked importance through three machine learning algorithms, Random Forest (RF), Support Vector Machine (SVM), and Least Absolute Shrinkage and Selection Operator (LASSO) methods. In addition, 100-time bootstrapped random sample process was used to make the SNPs ranking repeatable and reliable. Finally, two-model performance index, Area Under Curve (AUC), and accuracy were used to evaluate the minimum needed feature numbers and the best model in four machine learning models (RF, SVM, Decision Tree and Extreme Gradient Boosting). All machine learning analysis were completed by R version 3.5.3.

Protein interaction networking. To explore the possible mechanism and pathways associated with the SNP signatures linked to clopidogrel resistance, the five protein-coding genes from eight signature genes were subjected to protein–protein interaction analysis using the BioGRID database [38]. The graphical network is displayed and was created using the open-source software Cytoscape [39]).

**Figure 2:**
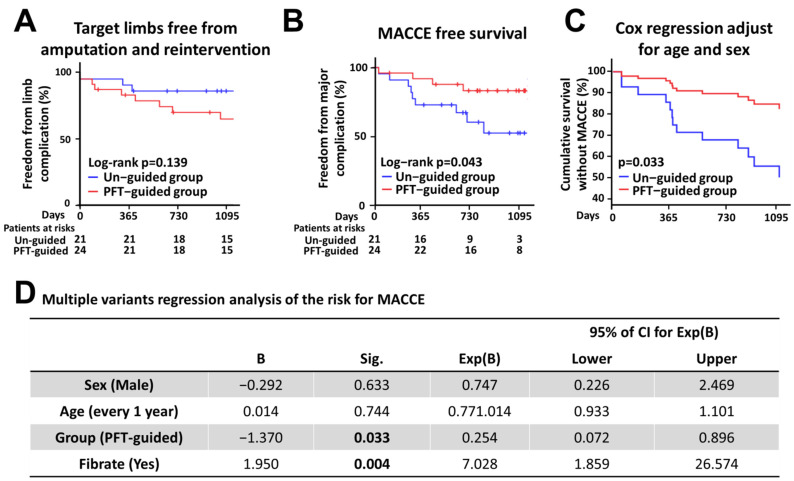
Kaplan–Meier analysis of the primary and secondary end points. (**A**,**B**) Shown is the percentage of patients who are free from the (**A**) primary end point, e.g., target limb amputation and reintervention; and (**B**) secondary end point, e.g., a composite of cardiovascular death, myocardial infarction, or ischemic stroke. Multiple variants regression analysis of the risk for MACCE. (**C**) Shown is the MACCE-free survival curve of patients in control and PFT-guided groups adjusted with age and sex. (**D**) Hazard ratio for MACCE among patients post-revascularization procedure, according to multi-variant Cox regression analysis. Variables were selected with a stepwise selection method. ALI, acute limb ischemia; Cre, creatinine; H/D, maintenance hemodialysis; PFT, platelet function test; DM, diabetes mellitus; COPD, chronic obstructive pulmonary disease; ABI, ankle-brachial index; Ac-GLU, fasting glucose; TG, triacylglycerol; HDL-C, high density lipoprotein-cholesterol.

**Figure 3:**
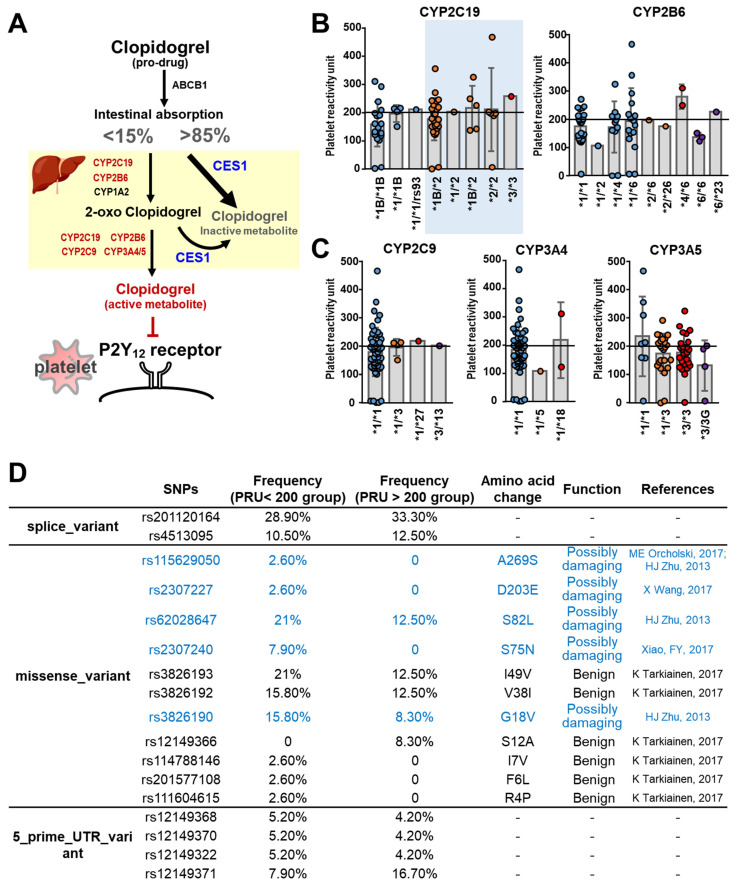
The SNPs in the pathways of clopidogrel metabolism. (**A**) Schematic illustration of the metabolic pathway of clopidogrel and the target receptors on platelets. (**B**) The platelet reactivity unit (PRU) in different genotypes of CYP2C19 and CYP6B6, the key enzymes convert clopidogrel into 2-oxo clopidogrel. (**C**) The PRU in different genotypes of CYP2C9 and CYP3A4/5, the key enzymes convert 2-oxo clopidogrel into activated form of clopidogrel. (**D**) The single-nucleotide polymorphisms (SNPs) of carboxylesterase 1 (CES1) and their functions. The figure is created with BioRender.com.

**Figure 4:**
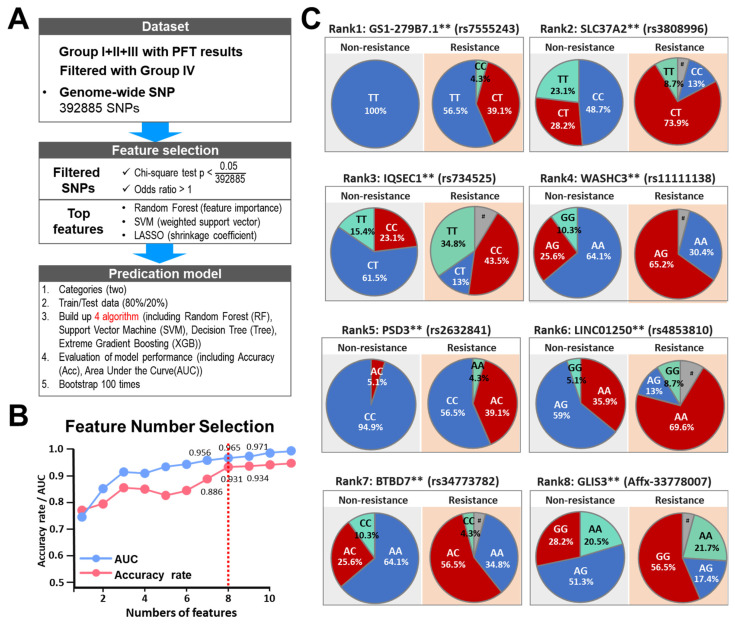
Prediction models built based on clinical features and genome-wide SNPs to classify clopidogrel-resistant patients using machine learning methods. (**A**) Machine learning workflow to select reliable and predictable features to build prediction models for group classification. (**B**) Accuracy and AUC of SVM model with different feature selection numbers. (**C**) Pie charts indicating the genotype frequencies of SNPs identified by AI-assisted analysis using SNP datasets obtained from patients of the four groups. # indicates the signaling of SNP array was lower than the calling rate. ** *p* < 0.005 by Chi-square test. # indicates that the signaling by the SNP array was lower than the calling rate. GS1-279B7.1 is annotated as a pseudogene. SLC37A2, Solute Carrier Family 37 Member 2; IQSEC1, IQ Motif Additionally, Sec7 Domain ArfGEF 1; PSD3, Pleckstrin Additionally, Sec7 Domain Containing 3; BTBD7, BTB Domain Containing 7; GLIS3, GLIS Family Zinc Finger 3.

Statistical analysis. All the data in this study are stratified according to the randomized group, presented as numbers with relative percentage or standard deviation. In Table 1 and Table 2, Figure 2 and Figure 3, fisher’s exact test was used to compare nominal variables, and the Mann–Whitney U-test was employed to compare continuous variables. The primary endpoint survival was calculated using Kaplan–Meier bivariable statistical analysis, the MACCE-free survival was calculated between the two groups, and the Kaplan–Meier curves were compared using log-rank test. Only those statistically significant variables in the univariant analyses were included in the multivariant analyses. To classify any independent factors affecting primary endpoint-analysis and MACCE time-to-event analysis while including a factor for the treatment group, stepwise regression analysis was performed using Cox multivariable proportional-hazards regression analysis. The statistically significant data were presented as adjusted Cox curve plots. The Chi-Square test was used to compare genotype frequencies distribution of SNPs between Non-resistance and resistance group in Figure 4. All statistics were performed using SPSS version 17 (SPSS Inc., Chicago, IL, USA). The *p* < 0.05 was indicated as the statistical significance.

## 3. Results

### 3.1. Subject Enrollment and Clinical Outcomes

From May 2016 through December 2017, a total of 600 diabetic patients, who were taking part in the NTCMRC, were screened for randomization. In total, 47 enrolled patients underwent randomization, and these individuals were followed until December 2019 to identify the occurrence of the composite primary events. At the completion of the trial, two patients (Group I) had withdrawn from the trial (Figure 1A). The median follow-up time was approximately 36 months. Another 19 diabetic PAD patients refused intervention (Group III) and 64 age-matched and sex-matched healthy participants (Group IV) were also included in the genomic SNP analysis.

#### 3.1.1. Baseline Characteristics

The baseline characteristics of the patients in the two groups are provided in Table 1. The baseline clinical characteristics are similar between the two groups, namely Group I and Group II. The medications used to treat these two groups were similar, except for a significant higher percentage of DDP4 prescriptions associated with the un-guided group (Group I).

#### 3.1.2. Platelet Reactivity

The baseline PFT values measured prior to the procedure are similar when the two groups are compared (251.3 ± 86.2 PRU in the PFT-guided group and 231.5 ± 83.4 PRU in the un-guided group). The mean values of PFT at the 36-month follow-up are significantly different when the two groups are compared (89.9 ± 77.5 PRU in PFT-guided group, and 180.8 ± 66.2 PRU in un-guided group; *p* = 0.005) (Table 2). There is also a significant difference when the changes in PFT values at baseline and at 36 months are compared for the clopidogrel-resistant patients treated with either ticagrelor or clopidogrel (Supplementary Figure S1; *p* = 0.0013). However, the difference in PFT values between the clopidogrel-resistant being treated with clopidogrel and clopidogrel-sensitive patients being treated with clopidogrel is not significant. 

#### 3.1.3. Clinical Outcomes

The post-revascularization 36-month ABI of the target limbs were significantly improved for both groups. The difference between the pre-revascularization and post-revascularization ABIs for the PFT-guided group seems to be higher than for the un-guided group; however, this was not statistically significant. Target limb revascularization was performed on two and three patients in the un-guided and PFT-guided groups, respectively, due to recurrence of intermittent claudication symptoms. Target limb amputation was performed on one and three of the patients in the un-guided and PFT-guided groups, respectively, due to severe tissue loss. During the follow-up, two patients died in each group (two from sudden death, one from cancer, and one due to heart failure after a CABG). Kaplan–Meier curves for the survival rates for target limb free from amputation and reintervention and all major adverse cardiovascular events are provided in Figure 2A,B. Notably, there are overt beneficial effects for the PFT-guided treatment with respect to a reduction in MACCE (Figure 2C). When the age- and sex-adjusted risk of MACCE is examined (all the baseline variables are included in the Cox regression analysis), the hazard ratio for MACCE in the PFT-guided group versus the un-guided group was −1.37 (95% CI, 0.072 to 0.896; *p* = 0.033). In addition, it seems that there is no interaction between the PFT-guided effect and any of the other variables. The results of the regression model for MACCE, taking into account the presence or absence of any major adverse cerebrovascular and cardiac events, are summarized in Figure 2D. The other factor that may affect the MACCE-free survival is fibrate medication. However, it is currently unknown whether fibrate medication increases the risk of MACCE; this will need further investigation and is beyond the scope of the present study. Information on the characteristics of the patients with and without fibrate medication is provided in Supplementary Table S1).

### 3.2. Pharmacogenetic Analyses of the Clopidogrel Metabolic Pathways

Clopidogrel is a prodrug that is absorbed in the small intestine and is activated in the liver by two enzymatic reaction steps to produce the active metabolite of clopidogrel: (1) first step is carried out by the enzymes CYP2C19, CYP2B6, and CYP1A2; (2) subsequently, the second step is brought about by the enzymes CYP2C19, CYP2C9, CYP2B6, and CYP3A4/5. The active metabolite then specifically and irreversibly binds to the P2Y12 receptors of platelets, which inhibits the aggregation of platelets, thereby preventing the formation of thrombi. On the other hand, after absorption by intestine, a significant proportion (>85%) of clopidogrel taken up by the patient undergoes extensive hydrolytic metabolism by the carboxylesterase 1 (CES1) in the liver into an inactive form. Accordingly, there are two catabolic pathways that compete for clopidogrel as a substrate (Figure 3A). We used whole-genome sequencing to investigate these pathways.

#### 3.2.1. Cytochrome P450

The genotypes of CYP2C19 alleles were examined in the 62 patients from Groups I, II, and III. Twenty-four patients (24/62, 38.7%) have the wild-type allele (CYP2C19 *1/*1), while the other thirty-eight patients (38/62, 61.3%) have a CYP2C19 genotype with either an allele resulting in decreased functionality (31/62, 50%; CYP2C19*1/*2 or *1/*3) or an allele resulting in loss-of-function (7/62, 11.3%; CYP2C19 *2/*2 or *3/*3). However, there is no significant difference in the PRU values of patients carrying the wild-type or loss-of-function alleles of CYP2C19 (Figure 3B; *p* = 0.2824). Regarding CYP3A4, there are only two patients (2/64, 3.1%) carrying a loss-of function allele (CYP3A4 *1/*18), while all the others carry the wild-type allele (CYP3A4 *1/*1). Furthermore, there is no correlation between the PRU values and the CYP2B6, CYP2C9, and CYP3A5 genotypes of patients (Figure 3B,C). 

#### 3.2.2. CES1

The dominant enzyme involved in the metabolism of clopidogrel in the liver is CES1, which catalyzes more than 85% of absorbed clopidogrel into inactive metabolites [40]. This means that less than 15% of clopidogrel is converted by cytochrome P450 into the active metabolite and is able to act to inhibit the P2Y12 receptor. There is a possibility that a decrease in CES1 activity might increase the available substrate (clopidogrel) and thus enhance the enzymatic conversion of clopidogrel into the active metabolite, thereby promoting anti-platelet activity. To study if CES1 has anything to do with the clopidogrel sensitivity in the patients, we analyze the CES1 genotypes of our subjects. Interestingly, our results indeed revealed that the frequency of CES1 SNPs that might damage enzymatic function seems to be higher in the clopidogrel-sensitive patients (PRU < 200) (Figure 3D). 

#### 3.2.3. P2Y12 Receptor

There were 83 SNPs identified in the 62 patients. All of these 83 SNPs were located in introns. None of the 9 SNPs present in these 62 patients reported to be pathogenic mutations are associated with clinical manifestations in the ClinVar (https://www.ncbi.nlm.nih.gov/clinvar/?term=P2RY12%5Bgene%5D, last access date 3 September 2021).

### 3.3. AI-Assisted Identification of SNP Signatures That Predispose towards Clopidogrel Resistance

To systemically identify the genetic factors that are associated with the clopidogrel resistance beyond cytochrome P450, and to explore the possible mechanism underlying this drug resistance phenotype, we carry out an AI-assisted whole-genome SNP association study. Patients from Group I, II, and III, as well as age-matched and sex-matched control subjects (Group IV, Figure 1B and Table 1), all from the NTCMRC took part in this aspect of the study. To identify SNPs, we genotyped the genomic DNA of all subjects using the AxiomTM Genome-Wide TWB 2.0 array plate [37], which contains 686,463 SNPs. SNPs with a minor allele frequency rate 0 and SNP missing rate larger than 10% were excluded from further analysis. At this point, there were 392,885 SNPs available for further analysis. First, the 392,885 SNPs were filtered using the χ^2^ test using a *p*-value < 0.05 and an odds ratio > 10; this left 422 significant SNPs as the top-ranked SNPs for further analysis. Patients were randomly assigned into training (80%) or validation (20%) sets and the ranking of these 422 significant SNPs were subject to three machine-learning methods (RF, SVM, LASSO). The above-mentioned process was repeated 100 times with bootstrapping of random combination of patients in the training and validation sets. The SNPs were ranked by the summarized counts using these 100 different combinations of samples obtained using the three machine learning methods. Finally, we build up four models (RF, SVM, LASSO, Decision Tree) to measure the best feature number using AUC and accuracy rate (Figure 4A). Among the top twenty SNPs selected (Supplementary Table S2), ten SNPs could be mapped to annotated genes and one SNP was mapped to a pseudogene. The top eight annotated SNPs were finally selected, and these had an AUC of 0.931 and accuracy rate of 0.965 (Supplementary Table S3; Figure 4B). 

Intriguingly, there are significant differences in the distribution of genotypes for all eight of these SNPs between the two groups of patients, namely the clopidogrel-resistant patients and non-resistant patients (Figure 4C), which suggests that these SNPs are located within or near to genetic factors associated with the pharmacogenetics of clopidogrel. Interestingly, four of the SNPs are intron variants of protein-encoding genes involved in the process of endocytosis, namely IQSEC1, WASHC3, PSD3, and BTBD7 (Supplementary Figure S2). Furthermore, two further SNPs are also intron variants of protein-encoding genes, namely GLIS3 and PRDM11; these genes have functions involved in the regulation of transcription. The remaining two SNPs are intron variants of a long non-coding RNA gene (LINC01250), and a protein encoding gene (SLC37A2) that is an antiporter for the transport of inorganic phosphate and glucose-6-phosphate. This information is summarized in Supplementary Table S3. 

**Figure 5:**
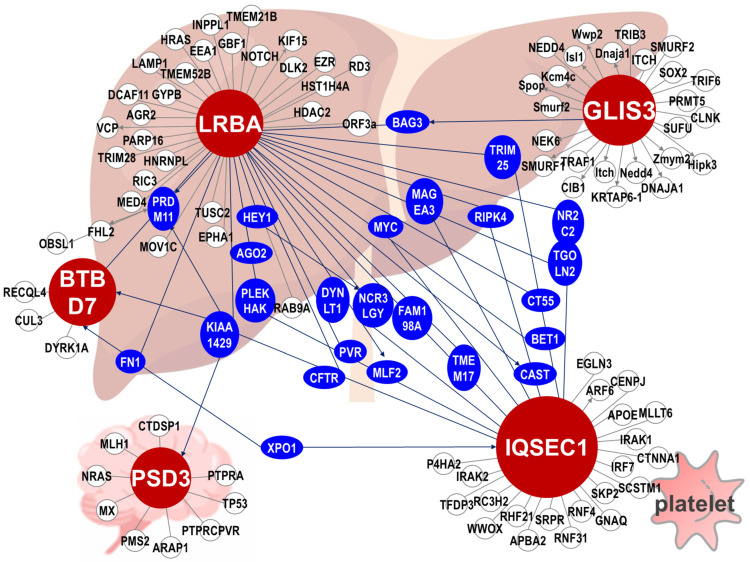
Protein–protein interaction network of the SNPs identified by AI-assisted analysis using SNP datasets obtained from patients of clopidogrel-resistant patients. The protein–protein interaction network was established by SNP within the protein coding genes. The red dots indicate the genes of AI-assisted SNP, and the blue dots indicate the proteins that connect the proteins in which the SNPs are located in the network. LRBA, LPS Responsive Beige-Like Anchor Protein. The figure is created with BioRender.com.

To explore if there is any relationship in terms of functional interaction among those top SNPs identified by AI-assisted methods, we perform a protein–protein interaction network analysis of the annotated genes where the SNPs are located. Remarkably, five of the proteins, namely IQSEC1, PSD3, BTBD7, GLIS3, and LRBA, are connected and form functional modules. Four of proteins (IQSEC1, PSD3, BTBD7, GLIS3) are in the list of the top eight SNPs (Supplementary Table S3; Figure 5), and one other protein (LRBA) is ranked seventeen in the list of top 20 SNPs (Supplementary Table S2).

## 4. Discussion

Several findings and discoveries can be pinpointed in the current study. Firstly, a surprisingly high proportion (>50%) of clopidogrel-resistant patients are found among diabetic PAD patients in Taiwan. Secondly, PFT-guided anti-platelet therapy does not reduce the chance of amputation or re-intervention of the target limbs in diabetic PAD patients. However, PFT-guided anti-platelet therapy is able to effectively reduce the occurrence of MACCE in diabetic PAD patients over the 3-year follow-up period. Furthermore, low-dose ticagrelor (90 mg per day) is able to effectively decrease PFT in clopidogrel-resistant patients when compared with the dosage recommended by other studies. Thirdly, AI-assisted genome-wide association study of a single nucleotide polymorphism (SNP) database identified an SNP signature composed of 20 SNPs, which are mapped into 9 protein-coding genes (SLC37A2, IQSEC1, WASHC3, PSD3, BTBD7, GLIS3, PRDM11, LRBA1 and CNR1). Finally, analysis of the protein connectivity map revealed that LRBA, GLIS3, BTBD7, IQSEC1, and PSD3 appear to form a protein interaction network. Intriguingly, the genetic factors seem to pinpoint a pathway related to endocytosis and recycling of P2Y12 receptor, which is the drug target of clopidogrel. Our findings reveal that a combination of AI-assisted discovery of SNP signatures and clinical parameters has a potential to develop an ethnic-specific precision medicine for antiplatelet therapy in diabetic PAD patients.

Dual antiplatelet therapy, namely aspirin combined with clopidogrel, has been proven to be effective in the management of patients with coronary artery disease [41], for the management of stroke patients with symptomatic large vessel high-grade intracranial atherosclerosis [42], and for stroke prevention among patients with atrial fibrillation [43]. In these patients, dual antiplatelet therapy provides clear benefits over aspirin monotherapy and is becoming the agent of choice for the prevention of thrombotic events. Dual antiplatelet therapy after intervention also improves outcomes among diabetic PAD patients [34]. In the REACH Registry, 40% of PAD patients experience a serious vascular event during 3-year follow-up despite current guideline-based medication [44]. The risk of recurrent vascular events is therefore high, particularly with some patients, specifically those with diffuse multivessel coronary artery disease, diabetes, recurrent myocardial infarction, peripheral artery disease, chronic kidney disease, etc. These guidelines also consider these patients to be at moderate to high risk of an ischemic event, and in the absence of a high risk of bleeding, dual antithrombotic therapy should be considered [45]. Currently, which drug, clopidogrel or ticagrelor, in combination with aspirin, will provide the best protection for diabetic PAD patients remains unknown.

### 4.1. The Prognosis of Clopidogrel-Resistant PAD Patients

The meta-analysis study published by Navarese et al. proved that dual antiplatelet therapy is able to significantly reduce the mortality of patients with PAD without increasing bleeding complications [7]. The prevalence of clopidogrel resistance, which is based largely on study populations made up mainly of Caucasian patients, had been reported to range from 28% to 39% [30,46]. However, clopidogrel resistance in Asian studies has been found to range from 36% to 58%, equivalent to the 57.8% in the present study [47,48]. This indicates that the difference in prevalence levels of high platelet activity after clopidogrel treatment needs to be carefully investigated because the above study populations are mainly composed of Caucasian patients with relatively few Asian patients. Furthermore, the majority of patients who participated in the studies that compared the therapeutic effects of DAPT on PAD were Caucasian (Supplementary Table S4) without diabetes [12,19,31,33,49,50,51,52,53]. The poor prognosis associated with atherosclerotic burden when diabetic patients are considered, when linked to the high prevalence of clopidogrel resistance, must significantly hinder the application of the findings used to develop the current guidelines. Importantly, in this context, our results provide a positive prospective trial of PFT-guided anti-platelet treatment for an Asian diabetic PAD patient populations [54].

During DAPT, whether PFT or the patient’s genotypes provide the information needed for patient management remains moot [55]. Patients with PAD have a lower response to DAPT, as well as a higher platelet reactivity, compared with patients with CAD [56]. Thus, stratification of clopidogrel-resistant PAD patients into those who need more intensive antiplatelet medications, such as ticagrelor, is an attractive option for this high-risk population [19]. Unfortunately, the EUCLID (Effects of Ticagrelor and Clopidogrel in Patients with Peripheral Artery Disease) trial demonstrated that monotherapy with ticagrelor is not superior to monotherapy with clopidogrel in terms of reducing the rate of cardiovascular events in patients with symptomatic PAD [12]. However, the results from the EUCLID trial may not be applicable to a high-risk diabetic PAD population such as the population forming our study. In the EUCLID trial, clopidogrel-resistant patients, who were homozygous for loss-of-function alleles, were excluded before randomization. In our study, those who were resistant to clopidogrel, including but not limited to homozygous for loss-of-function alleles, seem to be the ones that will benefit most from aspirin and ticagrelor combination therapy. Furthermore, only about 38% of the patients enrolled in the EUCLID trial were diabetic. Specifically, in the context of our study, the beneficial effects of ticagrelor have been proved to be significantly correlated with genotype [57]. The beneficial effect of ticagrelor vs. clopidogrel combined with aspirin observed in our trial provides additional insights into the need for further clinical tests that screen platelet activity in high-risk patients, such as diabetic PAD patients, who have had recent revascularization. Our results also demonstrate that, instead of CYP2C19 polymorphisms [58], a signature involving eight SNPs is able to predict clopidogrel resistance in these high-risk patients from Taiwan. Our results also highlight the need for a precision medicine approach to these patients that is linked to a personalized medical strategy, including in this case either PFT-guided therapy or genotype-guided therapy is used when deciding which anti-platelet medicine prescription is needed to provide cardiovascular and limb protection for PAD patients.

### 4.2. Ticagrelor Dosage

In the PEGASUS-TIMI 54 trial, a lower dosage of ticagrelor (60-mg twice daily) resulted in particularly favorable outcomes for CV and all-cause mortality among patients after myocardial infarction complicated with PAD [21]. Recently, a similar low dose (60 mg twice daily) of ticagrelor had the same effect on platelet activity reduction when compared with a higher dose (90 mg twice daily) [46]. A recent meta-analysis published by Chen et al. has also demonstrated that low-dose ticagrelor provided better cardiac protection when compared with clopidogrel when patients had a similar rate of bleeding complications [59]. From our results it can be seen that an even lower dose of ticagrelor, only 90 mg per day, is able to sustainably reduce the PFT of patients below 234 PRU and provide MACCE protection. Nonetheless, it should be noted that the lack of major hemorrhagic complications in our study might be related to either the small size of our cohort or to the very low dose of ticagrelor used. 

### 4.3. Cost-Effectiveness of Genotyping-Guided DAPT

Theidel et al. estimated that one-year treatment with ticagrelor was associated with an estimated 0.1796 life-years gained and a 0.1570 quality-adjusted life-years gained, respectively, over the lifetime horizon [60]. In Germany, a cost per additional life-year gained or quality-adjusted life-year in the range of EUR 25,000 (USD 33,000) to EUR 38,000 (USD 50,000) is generally considered cost-effective [61]. Comparing ticagrelor with the lowest priced generic clopidogrel, the incremental cost-effectiveness ratio was EUR 3118 per life-years gained (EUR 3567 per quality-adjusted life-years), which is significantly lower than the cost for life-year or quality-adjusted life-year gained [60]. Johnson et al. further concluded that the use of genotype-guided antiplatelet therapy to reserve prasugrel or ticagrelor use for patients with reduced CYP2C19 activity could prevent costs associated with adverse cardiac events [62]. The total cost of genotyping using TWB 2.0 and PRU test in Taiwan is around USD 180 per patient. Compared with the cost of possible morbidity and MACCE, USD 180 is an acceptable expense to provide personalized DAPT for post-interventional diabetic PAD patients.

### 4.4. Precision Medicine for DAPT: Resensitization of the P2Y12 Receptor

CYP2C19 polymorphisms, especially loss-of-function alleles, play an important role in clopidogrel resistance, but there remain additional genetic variants that need to be identified [16]. Frelinger et al. reported that all known genetic and nongenetic factors together accounted for only 18% of the pharmacokinetic variation and between 32% and 64% of the clopidogrel pharmacodynamic variation [63]. Results from other studies have also demonstrated that the CYP2C19 genotype alone or the CYP2C19 genotype combined with clinical variables is only able to explain either 5% or 11% to 20% of the variability in platelet reactivity, respectively [16]. Furthermore, the results from Nasyuhana Sani et al. and this study together confirm that clopidogrel nonresponders can be found not only in patients with heterozygous or homozygous CYP2C19 loss-of-function alleles but also in patients without a LOF allele [64]. These findings suggest that polymorphisms affecting cytochrome p450, including CYP2C19 and a number of other alleles, contribute to only a portion of the variability in the antiplatelet effect of clopidogrel when diverse patient populations are considered [16]. Novel genetic variants that affect the clopidogrel response, beyond the well-known CYP2C19 LOF alleles remain to be identified. 

Adenosine diphosphate (ADP) mediates its actions through G-protein-coupled receptors and the purinoceptors P2Y1 and P2Y12 when triggering platelet activation [65]. The activities of the purinoceptors are rapidly and reversibly modulated in human platelets, with the underlying mechanism including receptor internalization and subsequent trafficking. The responsiveness of P2Y12 receptors in human platelets is rapidly desensitized when there is exposure to ADP and rapidly resensitized upon removal of ADP [66]. Activation of the P2Y12 receptors stimulates ADP ribosylation factor 6 (ARF6) activity, which facilitates dynamin-dependent fission of clathrin-coated vesicles, and there is subsequently internalization, which is an essential factor for platelet activation [67]. Blockade of receptor internalization or subsequent recycling by specific mutations will diminish receptor resensitization within the human platelets of individuals with bleeding disorders [66]. Our results have identified four SNPs (IQSEC1, WASHC3, PSD3, BTBD7) that are involved in the process of endocytosis. IQSEC1 has been shown to act at clathrin-coated pits to promote integrin internalization and clathrin-mediated endocytosis [68]. PSD3 associated with DYN2 is able to activate ARF6 [69]. Btbd7 is able to induce loss of E-cadherin and increased cleft formation [70]. The WASH complex promotes actin nucleation, facilitates endocytosis and increases intracellular membrane transport [71]. 

### 4.5. Limitations

This is a single-center study, underpowered in terms of clinical events and probably not large enough to provide full insight into the correlation of clopidogrel resistance with MACCE in diabetic patients who have PAD complications. The small sample size of participants in this study may create a risk of selection bias and need a larger cohort for validation. In addition, other interventional options, such as endovascular intervention or autologous venous graft bypass for lesion >4 cm, might affect long-term patency and amputation-free survival. In addition, future studies need to investigate in more detail whether ticagrelor, at a dosage of 90 mg per day, is able to reduce MACCE among clopidogrel-resistant diabetic patients complicated with PAD.

## 5. Conclusions

PFT-guided DAPT appears to reduce MACCE in diabetic patients with PAD complications post-revascularization. However, the regimen is not able to reduce the rate of target-limb reintervention or amputation. The low dosage of ticagrelor used in our study, together with the high prevalence of clopidogrel resistance in our study population, has allowed us to demonstrate that a PFT-guided precise personal anti-platelet regimen is needed when the target population has a high proportion of clopidogrel resistance. The differences in genetic background between Caucasian and Asian populations demands that previous studies need to be carefully applied to patients of Asian origin. Our findings provide additional insights into the possible genetic mechanism(s) behind clopidogrel resistance in humans and into the prognosis of clopidogrel-resistant diabetic patients with PAD after revascularization. Our findings highlight the need for future studies; their aim should be the development of a precision personal antiplatelet regimen that can be used individually on diabetic patients after revascularization for PAD.

## Figures and Tables

**Table 1:** Baseline characteristics of diabetic patients with peripheral artery disease who were randomly assigned into the un-guided (Group I), the PFT-guided (Group II) group and those who refused intervention (Group III). These are compared with age- and sex-matched healthy participants (Group IV) recruited from the Northeastern Taiwan Community Medicine Research Cohort.

Group	I	II	I vs. II	III	IV
	Un-guided (n = 21)	PFT-guided (n = 24)	*p*	Medical (n = 19)	Control (n = 64)
**Sex, male** (n, %)	11(52.4%)	16 (66.7%)	0.15	15 (78.9%)	42 (65.6%)
**Age** (years)	63.0 ± 7.4	63.9 ± 6.3	0.44	63.7 ± 11.0	63.8 ± 13.3
**Body weight** (Kg)	68.9 ± 10.2	68.4 ± 15.4	0.60	63.2 ± 10.6	57.9 ± 6.7
**Ejection fraction** (%)	65.4 ± 8.5	69.0 ± 6.8	0.23	N/A	N/A
**Smoking** (%)			0.336		
Current	12 (57.1%)	13 (54.2%)		7 (36.8%)	28 (43.8%)
None	7 (33.3%)	5 (20.8%)		12 (63.2%)	36 (56.2%)
Quit	2 (9.5%)	6 (25.0%)			
**Co-morbidity**					
DM	21 (100%)	24 (100%)	1.00	19 (100%)	0
Hypertension	15 (71.4%)	15 (62.5%)	0.32	12 (63.2%)	0
CAD	6 (28.6%)	7 (29.2%)	0.54	10 (52.6%)	0
Old CVA	3 (14.3%)	2 (8.3%)	0.27	2 (10.5%)	0
CKD stage 2–3	3 (14.3%)	4 (16.7%)	0.50	2 (10.5%)	0
**Medications**					
Aspirin	16 (76.2%)	10 (41.7%)	0.034	13 (68.4%)	0
Cilostazol	6 (28.6%)	8 (33.3%)	0.759	0 (0%)	0
Pentoxifylline	3 (14.3%)	6 (25.0%)	0.469	0 (0%)	0
Acrabose	3 (14.3%)	4 (16.7%)	1.0	3 (15.8%)	0
Thiazolidinedione	0 (0%)	2 (8.3%)	0.491	1 (5.3%)	0
DPP4	15 (71.4%)	9 (37.5%)	**0.036**	3 (15.8%)	0
Sulfonylurea	11(52.4%)	16 (66.7%)	0.374	4 (21.1%)	0
Metformin	17 (81.0%)	14 (58.3%)	0.121	5 (26.3%)	0
SGLT2 inhibitor	2 (9.5%)	2 (8.3%)	1.0	6 (31.6%)	0
Diuretics	6 (28.6%)	8 (33.3%)	0.759	3 (15.8 %)	0
CCA	12 (57.1%)	14 (58.3%)	1.0	6 (31.6%)	0
Beta-blocker	11 (52.4%)	15 (62.5%)	0.555	10 (52.6%)	0
ACEI	2 (9.5%)	3 (12.5%)	1.0	2 (10.5%)	0
ARB	16 (76.2%)	15 (62.5%)	0.356	14 (73.7%)	0
Nitrate	3 (14.3%)	1 (4.2%)	0.326	6 (31.6%)	0
Statin	15 (71.4%)	21 (87.5%)	0.267	11 (57.9%)	0
Fibrate	3 (14.3%)	3 (12.5%)	1.0	1 (5.3%)	0
Ezetimibe	5 (23.8%)	5 (20.8%)	1.0	2 (10.5%)	0
**Biochemistry data**					
Total cholesterol (mg/dL)	154.4 ± 28.8	160.5 ± 44.7	0.94	150.6 ± 26.4	199.7 ± 35.3
HDL (mg/dL)	39.1 ± 11.6	42.2 ± 13.7	0.35	41.3 ± 9.4	62.8 ± 14.3
LDL (mg/dL)	80.9 ± 23.9	92.3 ± 42.8	0.53	92.3 ± 24.5	126.7 ± 30.7
Triglyceride (mg/dL)	204.8 ± 126.5	149 ± 63.5	0.112	158.6 ± 100.4	96.9 ± 29.9
Glycohemoglobin (%)	7.99 ± 1.43	8.10 ± 1.82	0.84	7.24 ± 1.51	5.49 ± 0.31
Fasting sugar (mg/dL)	143.8 ± 39.3	140.7 ± 49.6	0.957	141.1 ± 60.9	90.0 ± 5.0
Creatinine (mg/dL)	1.22 ± 0.54	1.66 ± 0.77	0.063	0.96 ± 0.29	0.87 ± 0.15
ALT (U/L)	22.5 ± 12.1	23.5 ± 16.1	0.92	22.4 ± 9.6	22.1 ± 5.9

Values are mean ± SD or n (%). DM, diabetes mellitus; CAD, coronary artery disease; CVA, cerebrovascular disease; CKD, chronic kidney disease; CCA, calcium channel blocker; ACEI, angiotensin-converting enzyme inhibitor; ARB, angiotensin II receptor blockers; DPP4, Dipeptidyl peptidase 4; SGLT2, sodium/glucose cotransporter 2; HDL, high-density lipoproteins; LDL, low-density lipoproteins; ALT, Alanine Transaminase; PFT, platelet function test.

**Table 2:** PFT-guided dual anti-platelet treatment prevents MACCE in diabetic PAD patients.

Group	Un-Guided (n = 21)	PFT-Guided (n = 24)	*p*
PFT at baseline (PRU)	231.5 ± 83.4	251.3 ± 86.2	0.937
Clopidogrel resistance PRU > 234 (n, %)	12 (57.1%)	14 (58.3%)	0.53
PFT at 36 months (PRU)	180.8 ± 66.2	89.9 ± 77.5	**0.005**
**Revascularization procedures**			
Bilateral (n, %)	10 (27.6%)	17 (70.8%)	0.09
Right only (n, %)	6 (28.6%)	5 (20.8%)	
Left only (n, %)	5 (23.8%%)	2 (8.3%)	
ABI at baseline	0.84 ± 0.17	0.76 ± 0.15	0.05
ABI at 36 months	0.92 ± 0.15*	0.89 ± 0.20	0.61
Paired T test of ABI (pre-op vs. post-op)	***p* = 0.03**	***p* = 0.0006**	
Difference of ABI (%)	14% ± 30%	21% ± 29%	0.28
**Primary Endpoint**			
Re-intervention	2 (9.5%)	3 (12.5%)	0.47
Amputation	1 (4.8%)	3 (12.5%)	0.52
Hemorrhagic episode	0 (0%)	0 (0%)	ns
**Secondary Endpoint**			
MACCE (n, %)	10 (47.6%)	6 (25.0%)	**0.02**
Death (n, %)	2 (9.5%)	2 (8.3%)	0.24
Ischemic CVA (n, %)	4 (19.0%)	2 (8.3%)	0.08
AMI (n, %)	5 (23.8%)	2 (8.3%)	**0.01**

Values are mean ± SD or n (%). ABI, ankle-brachial index; CVA, cerebrovascular disease; PFT, platelet function test; MACCE, major adverse cerebrovascular and cardiac events; AMI, Acute myocardial infarction; PRU, Platelet (P2Y_12_) reaction units.

## Data Availability

The data presented in this study are available on request from the corresponding author. The data are not publicly available due to ethical regulation.

## Supplementary Materials

The following are available online at https://www.mdpi.com/article/10.3390/biomedicines10010116/s1, Figure S1: The differences in PFT after 36-month ticagrelor or clopidogrel treatment in clopidogrel-sensitive and -resistant patients; Figure S2: The potential pathway of P2Y 12 receptors recycling by endocytosis; Table S1: Characteristics of patients with or without fibrate medication; Table S2: Top 20 important features; Table S3: Top 8 annotated genes and the functions; Table S4: Under-representative Asian and diabetic population in PAD studies.

## Abbreviations

ABI	Ankle-brachial index
ACT	Activated clotting time
AI	Artificial intelligence
AUC	Area under curve
CES1	Carboxylesterase 1
DAPT	Dual anti-platelet therapy
EUCLID	Effects of Ticagrelor and Clopidogrel in Patients with Peripheral Artery Disease
IRB	Institutional Review Board
LASSO	Least Absolute Shrinkage and Selection Operator
MACCEs	Major adverse cerebrovascular and cardiac events
NTCMRC	Northeastern Taiwan Community Medicine Research Cohort
PAD	Peripheral artery disease
PFT	Platelet function test
PRU	Platelet reactivity in units
RF	Random Forest
SNP	Single-nucleotide polymorphism
SVM	Support Vector Machine
