# Supplementary material for: Artificial-Intelligence-Assisted Discovery of Genetic Factors for Precision Medicine of Antiplatelet Therapy in Diabetic Peripheral Artery Disease
Source: Biomedicines. 2022 Jan 6;10(1):116. doi: 10.3390/biomedicines10010116 (PMC8773099; doi:10.3390/biomedicines10010116)
Supplement: Supplementary file 1 [file biomedicines-10-00116-s001.zip › biomedicines-1518797-supplementary.pdf]

Supplementary information

Artificial intelligence-assisted discovery of genetic factors for precision medicine of antiplatelet therapy in diabetic peripheral artery disease

Chi-Hsiao Yeh[1,2,3], Yi-Ju Chou[4], Tsung-Hisen Tsai[5], Paul Wei-Che Hsu[4], Chun-Hsien Li[5], Yun-Hsuan Chan[5], Shih-Feng Tsai[4], Soh-Chin Ng[6], Kuei-Mei Chou[6], Yu-Ching Lin[2,7], Yu-Hsiang Juan[2,7], Tieh-Cheng Fu[2,8], Chi-Chun Lai[2,3,9]*, Huey-Kang Sytwu[10,11]*, Ting-Fen Tsai[4,12,13]*

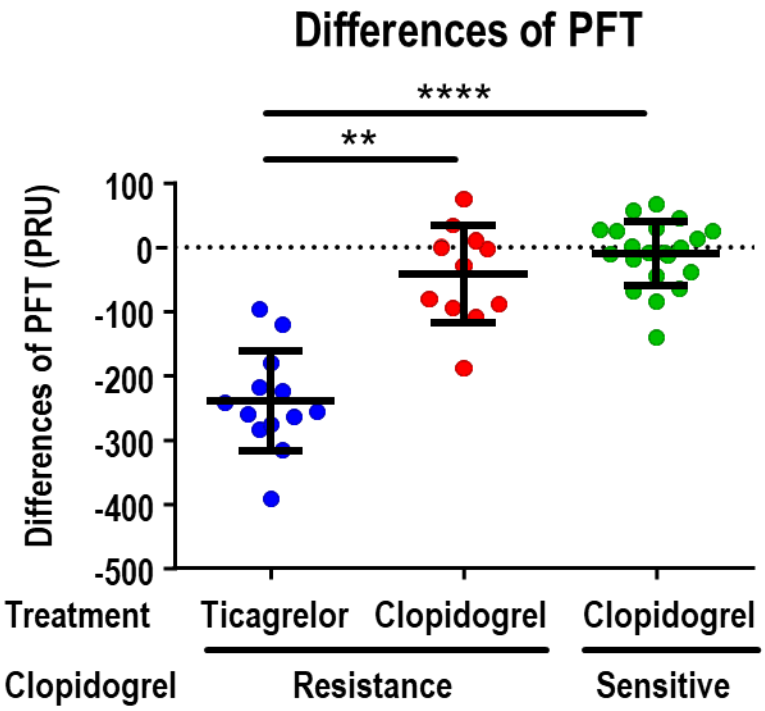

Supplementary Figure S1. The differences of PFT after 36-month ticagrelor or clopidogrel treatment in clopidogrel sensitive and resistant patients. PFT, platelet function test; PRU, platelet reactivity unit. **, p<0.01 ****, p<0.0001

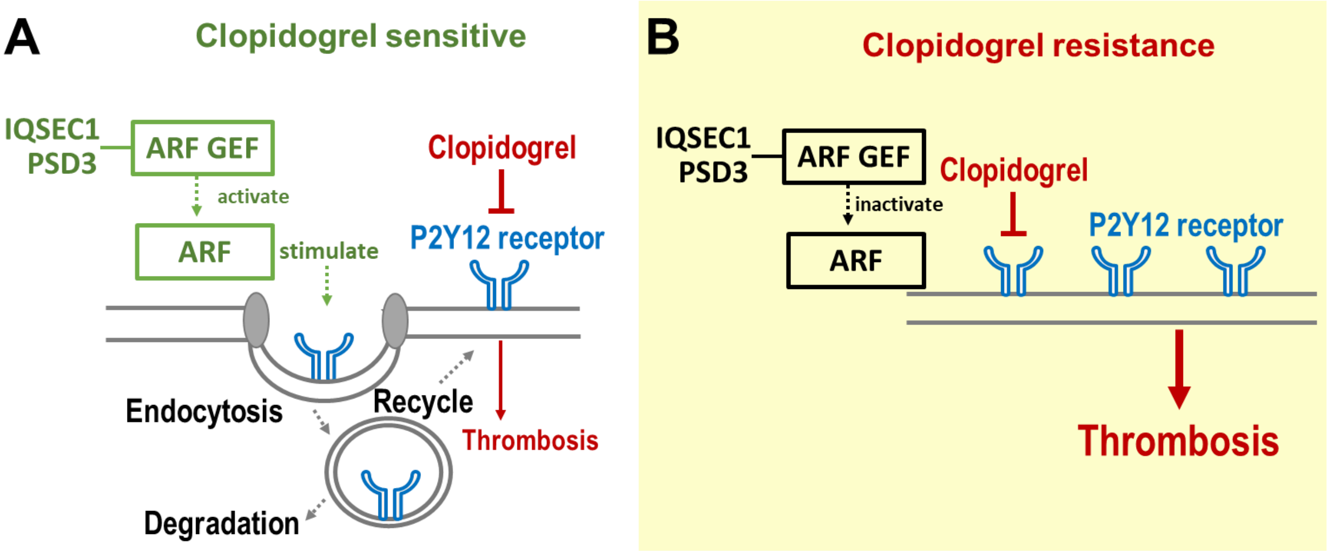

Supplement Figure S2. The potential pathway of $P2Y_{12}$ receptors recycling by endocytosis.(A) Activation of the P2Y12 receptors stimulates ADP ribosylation factor 6 (ARF6) activity, which facilitates dynamin-dependent fission of clathrin-coated vesicles and subsequently there is internalization, which is an essential factor needed for platelet activation. Clopidogrel acts as antagonists of $P2Y_{12}$ to prevent thrombosis. (B) A Blockade of receptor internalization or subsequent recycling by specific mutations will diminish receptor resensitization within the human platelets of individuals with bleeding disorders. Furthermore, the efficiency of clopidogrel may be deceased under this situation.

Supplementary Table S1. Characteristics of patients with or without fibrate medication

Fibrate	No (n=39)	Yes (n=6)	p
**Age (year)**	65.0 ± 6.7	65.0 ± 8.1	.352
**Body weight (Kg)**	67.3 ± 12.4	77.7 ± 15.0	.400
**HbA1c (%)**	8.0 ± 1.6	8.1 ± 1.7	.961
**PFT at baseline(PRU)**	237.5 ± 79.7	271.5 ± 115.9	.095
**Creatinine (mg/dL)**	1.4 ± 0.7	1.9 ± 0.3	.101
**LDL (mg/dL)**	87.4 ± 37.7	85.8 ± 20.2	.530
**Total Cholesterol (mg/dL)**	156.0 ± 39.8	168.3 ± 18.9	.274
**Triglyceride (mg/dL)**	173.5 ± 99.8	188.8 ± 120.7	.308
**CVA (n, %)**	5 (12.8%)	0 (0%)	1.0
**CAD (n, %)**	12 (30.8%)	1 (16.7%)	.656
**Statin (n, %)**	33 (84.6%)	3 (50.0%)	.084
**Ezetimibe (n, %)**	8 (20.5%)	2 (33.3%)	.601

Values are mean ± SD or n (%). CAD, Coronary artery disease; CVA, cerebrovascular disease; PFT, platelet function test; PRU, Platelet ($P2Y_{12}$) reaction units.

Supplementary Table S2. Top 20 important features

Rank	Feature (SNP)	Count	position	gene	Protein Connectivity map
1	rs10918666	222	chr1:167208924	None	
2	rs7555243	209	chr1:185325621	GS1-279B7.1: Intron Variant	
3	rs3808996	156	chr11:125086402	SLC37A2: Intron Variant	
4	rs734525	135	chr3:12907528	IQSEC1: Intron Variant; LOC105376956: Intron Variant	V
5	rs11111138	122	chr12:102027512	WASHC3: Intron Variant	
6	rs2632841	118	chr8:18814273	PSD3: Intron Variant	V
7	rs4853810	113	chr2:2936195	LINC01250: Intron Variant	
8	rs1917098	100	chr3: 78255075	None	
9	rs9650076	99	chr8: 119121033	None	
10	rs6030731	87	chr20: 43252271	None	
11	rs9576776	86	chr13: 39321269	None	
12	rs6922617	84	chr6: 41368386	None	
13	rs34773782	80	chr14:93248234	BTBD7: Intron Variant	V
14	Affx-33778007	78	chr9:4036449	GLIS3: Intron Variant	V
15	rs3758726	76	chr11:45220030	PRDM11: Intron Variant	
16	rs74834148	76	chr7: 56257428	None	
17	Affx-23639462	73	chr4: 150375232	LRBA: Intron Variant	V
18	rs6691208	72	chr1: 3014763	None	
19	rs6454674	70	chr6: 88163211	CNR1: Intron Variant	
20	rs7545995	70	chr1: 3053769	None	

Supplementary Table S3. Top 8 annotated genes and the functions

Rank	SNP	gene	Expression of organ	Function
1	rs3808996	SLC37A2	Proximal GI tract, bone marrow and lymphoid tissues	Inorganic phosphate and glucose-6-phosphate antiporter.
2	rs734525	IQSEC1	All tissues	control of vesicle formation by endocytosis cargo
3	rs11111138	WASHC3	All tissues, especially smooth muscle and epithelial tissues.	nucleation-promoting factor (NPF) at the surface of endosomes
4	rs2632841	PSD3	All tissues, especially brain	Endocytosis
5	rs4853810	LINC01250		Long non-coding RNA
6	rs34773782	BTBD7	All tissues	mediator of epithelial dynamics and organ branching by promoting cleft progression
7	Affx-33778007	GLIS3	All tissues	repressor and activator of transcription
8	rs3758726	PRDM11	All tissues	involved in transcription regulation

Supplementary Table S4.

First Author (year)	Type	Patient population	intervention	Subgroups Reported	Ischemic Outcome	Platelet reactivity (% of resistance)	Maximum FU (months)	Mean Age (yrs)	DM (%)	Asian patients % (n)
Hiatt et al (2017)[69]	RCT (EUCLID)	Symptomatic PAD	Ticagrelor (90 mg bid) vs Clopidogrel (75 mg QD)	CYP2C19, exclude poor, LOF (42.1%) and non-LOF	MACE	N/A	36	66	38.5%	11.5% (1602/13885)
Patel et al (2020)[51]	RCT (Post-hoc analysis of PLATO)	ACS+PAD	Aspirin/Ticagrelor (90 mg bid) vs Aspirin /Clopidogrel (75 mg QD)	None	MACE	N/A	12	66	37.7%	<4.5% non-Caucasian (51/1144)
Bonaca et al (2016)[48]	RCT (PEGASUS-TIMI 54)	MI+PAD	Aspirin/Ticagrelor (90 mg or 60 mg bid) vs Aspirin	none	MACE	N/A	36	66	42.17%	<4.3% (49/1143)
Ducci et al (2020)[19]	RCT	PAD+stent	Aspirin/Ticagrelor (90 mg bid) vs Aspirin /Clopidogrel (75 mg QD)		Target limb revascularization	PFT (by PRU)	12	71-74	67.5%	N/A (40)
Yang et al (2018)[52]	RCT	PAD+stent	Aspirin/Ticagrelor (90 mg bid) vs Aspirin / Clopidogrel (75 mg QD)	N/A	white thrombus	N/A	6	60-71	50%	N/A (18)
Lee et al. (2019)[30]	Retrospective	PAD+stent	Clopidogrel (75 mg QD)	LOF (44.9%) and non-LOF	Amputation free survival	VerifyNow (55.8%)	12	72	76.9%	100% (278)
Guo et al. (2014)[49]	observational	PAD+endovascular	Aspirin (100 mg QD)/ Clopidogrel (75	LOF (52%) and non-LOF		N/A	12	73-74	42%	100% (50)

		therapy	mg QD)							
Spiliopoulos et al (2013)[32]	observational	PAD+endovascular therapy	Aspirin (100 mg QD)/ Clopidogrel (75 mg QD)	N/A	death, bleeding, major amputation, or TLR	N/A	12	68.5	57%	N/A (100)
Pastromas et al (2013)[50]	observational	PAD+endovascular therapy	Aspirin (100 mg QD)/ Clopidogrel (75 mg QD)	N/A	TLR-free survival	N/A	96 (mean 27.7+22.9)	69	56.6%	N/A (113)
